# Dream Recall/Affect and Cortisol: An Exploratory Study

**DOI:** 10.3390/clockssleep4010003

**Published:** 2022-01-29

**Authors:** Alexandros S. Triantafyllou, Ioannis Ilias, Nicholas-Tiberio Economou, Athina Pappa, Eftychia Koukkou, Paschalis Steiropoulos

**Affiliations:** 1 MSc Program in Sleep Medicine, Medical School, Democritus University of Thrace, 68100 Alexandroupolis, Greece; alexandros.st.triantafyllou@gmail.com (A.S.T.); nt_economou@yahoo.it (N.-T.E.); steiropoulos@yahoo.com (P.S.); 2 Department of Endocrinology, Diabetes and Metabolism, Elena Venizelou Hospital, 11521 Athens, Greece; athpappa@gmail.com (A.P.); ekoukkou@gmail.com (E.K.); 3 Sleep Study Unit, Eginition Hospital, University of Athens & Enypnion Sleep Disorders—Epilepsy Center, 11521 Athens, Greece; 4 Department of Pneumonology, Medical School, Democritus University of Thrace, 68100 Alexandroupolis, Greece

**Keywords:** dreams, dream recall, dream affect, cortisol, menstrual cycle

## Abstract

The effect of cortisol on dreams has been scarcely studied. The aim of this exploratory study was to assess the possible effect of cortisol levels on dream recall/affect, considering, in female subjects, their menstrual cycle phase. Fifteen men and fifteen women were recruited. Saliva samples were used for the detection of cortisol levels. Participants were instructed to provide four saliva samples, during three consecutive days. After awakening, on the second and third day, they were asked whether they could recall the previous night’s dreams and whether these were pleasant or unpleasant. Female subjects followed this procedure twice: firstly, during the luteal phase and, secondly, during the follicular phase of the menstrual cycle. Subjects with higher evening or higher morning cortisol levels tended to show increased dream recall; a non-statistically significant association between morning cortisol levels and positive dream affect was also found. This association acquired statistical significance for salivary morning cortisol levels exceeding the upper normal level of 19.1 nmol/L (OR: 4.444, 95% CI: 1.108–17.830, *p*-value: 0.039). No connection between menstrual cycle stages and dream recall/affect was detected. In conclusion, cortisol may be a crucial neuromodulator, affecting dream recall and content. Therefore, its effects on sleep and dreams should be further studied.

## 1. Introduction

Dreams constitute a universal phenomenon of human sleep. The ability, however, to recall a dream varies among individuals [1,2]. The emotional quality of dreams also differs from person to person, as well as from night to night. The processes affecting these phenomena remain obscure; nevertheless, several agents have been proposed to influence dream recall and affect [3,4,5].

Glucocorticoids and especially cortisol may play a fundamental role in the aforementioned procedures [6]. Cortisol, normally, follows a circadian pattern, showing its highest concentration several minutes after awakening and its lowest in the evening [7]. It has been suggested that dream formation and memory consolidation share common biological processes, in which cortisol may be the key element [8,9]. Cortisol receptors are distributed throughout the brain and especially the limbic system and hippocampus [10]. The function of hippocampus is crucial for dream formation and dream recall [11]. Therefore, cortisol affecting hippocampus, may influence the processes of memory consolidation and eventually dream recall [12]. The effect of cortisol, however, on hippocampus seems to be biphasic, following an inverted U-shaped model [8]. More specifically, lower levels of cortisol may enhance the excitability of hippocampus, whereas, when cortisol values exceed a threshold, this may have the opposite effect. Moreover, cortisol levels peak during the Rapid Eye Movement (REM) stage of sleep [9]. Dream recall has been noted to be increased for REM dreams [3].

Cortisol has been proposed to affect the nature of dreams by altering the communication of hippocampus with neocortex [9]. This communication is crucial for memory consolidation and therefore for dream formation. When this communication is disrupted, by high cortisol levels, only fragments of dreams may be generated. Thereafter, “malfunctioning” neocortex and hippocampus struggle to construct a narrative based on these fragments, which eventually may feel bizarre. This process may continue after awakening, when cortisol levels keep rising.

Moreover, several studies have shown that cortisol levels are associated with dream recall and content. Sufferers from frequent nightmares have been reported to express increased cortisol levels after nightmares [13]. Blunted cortisol awakening response (CAR) has been associated with frequent nightmares [14,15]. Patients suffering from Cushing’s syndrome may report frequent dreams with bizarre and vivid content [16]. Depression has been linked to dysregulation of the Hypothalamic–Pituitary–Adrenal (HPA) axis and increased cortisol levels [17]. This phenomenon may explain the increased frequency of sleep dysfunctions and nightmares in these patients [18]. On the other hand, patients with post-traumatic stress disorder (PTSD), who suffer from frequent nightmares, are characterized by decreased cortisol levels compared to healthy subjects [6]. In a previous study of ours, women reported more frequently pleasant dreams during the luteal phase of the menstrual cycle, where cortisol levels are slightly lower compared to the follicular phase [19].

In this context, in this study, we primarily aimed to explore the possible association between cortisol levels and dream recall/affect, taking into account in women participants their menstrual cycle phase.

## 2. Results

Cortisol levels, as well as dream recall/affect, in male and female participants, are presented in Table 1.

**Figure 1:**
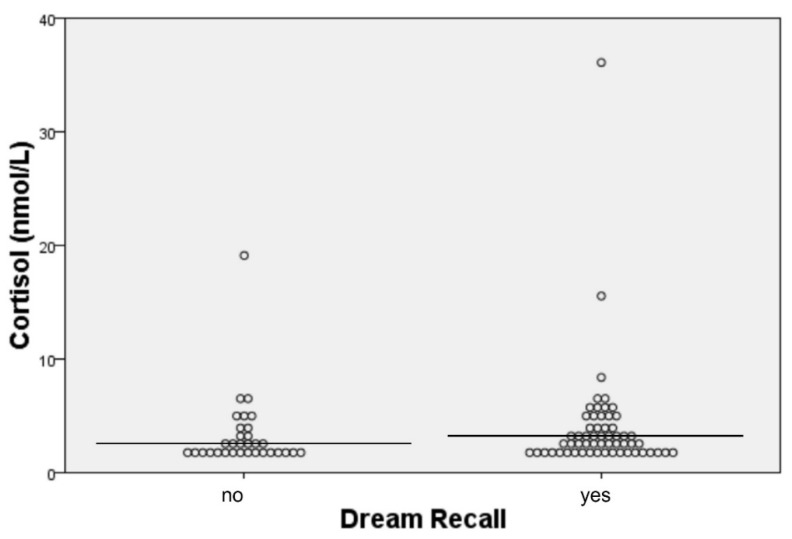
Dot plot depicting the association of cortisol levels, before sleep, with dream recall.

**Figure 2:**
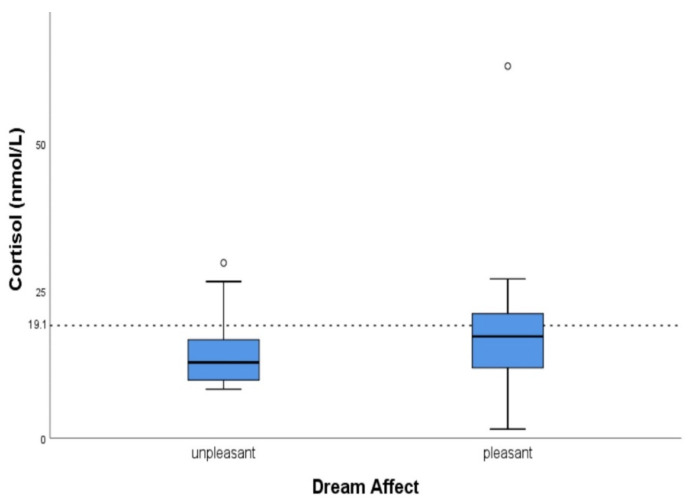
Box plot presenting the association of cortisol levels, after awakening, with dream affect. Horizontal lines represent the median cortisol values for each group. Hollow circles represent the outlier values.

**Figure 3:**
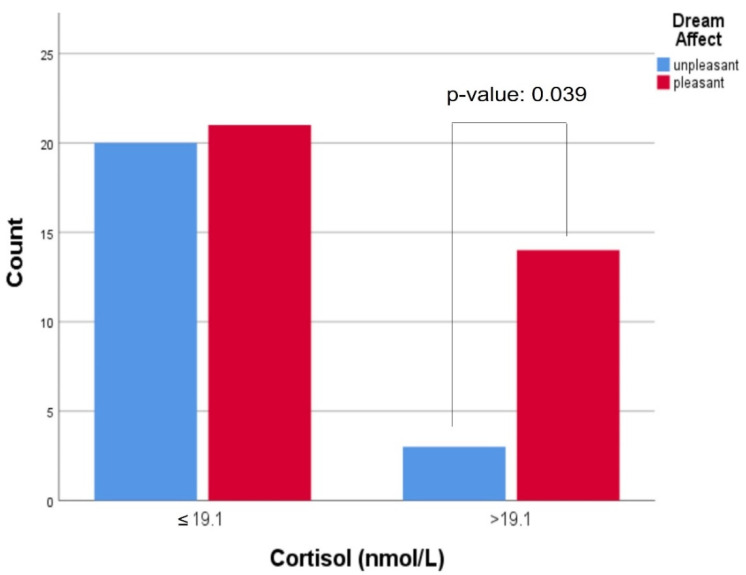
Clustered bar count of a morning cortisol cut-off value of 19.1 nmol/L by dream affect, *p*-value: 0.039.

Regarding the possible connection between cortisol levels and dream recall/affect, no statistically significant results were found, when each group of participants was studied separately, for each individual sampling day. However, when all participants were studied together, for all sampling days collectively, higher levels of cortisol, before sleep, tended to be associated with enhanced dream recall (OR: 1.052, 95% CI: 0.919–1.203, *p*-value: 0.141) (Figure 1). Participants with higher morning cortisol levels tended to describe their dreams as pleasant (OR: 1.054, 95% CI: 0.962–1.154, *p*-value: 0.098) (Figure 2). More specifically, morning cortisol levels, exceeding the value of 19.1 nmol/L (upper normal cortisol limit after awakening [20]), were associated with pleasant dream affect in a statistically significant manner (OR: 4.444, 95% CI: 1.108–17.830, *p*-value: 0.039) (Figure 3). No other associations between cortisol levels and dream recall/affect were noted. Implementing mixed model analysis, more frequent dream recall was also noted with higher morning salivary cortisol (F (1.570) = 7.299, *p* = 0.037, eta^2^ = 0.021); no differences in affect vis à vis morning salivary cortisol were noted with this analysis. Moreover, evening salivary cortisol levels (after inverse (1/x) transformation) did not show differences vis à vis dream recall or affect with mixed model analysis (all *p* > 0.1).

Finally, we found no statistically significant connection between the stage of menstrual cycle and dream recall/affect for each day separately (D2a vs. D2b, D3a vs. D3b), as well as regardless of the sampling day (D2a + D3a vs. D2b + D3b).

## 3. Discussion

In this study, our results point—albeit tentatively—towards better dream recall when cortisol levels are increased before and after sleep. To the best of our understanding, this study is the first to exhibit an association between increased cortisol levels before sleep and enhanced dream recall. Our study has also demonstrated that higher cortisol levels after awakening tend to affect dream content in a positive way (Figure 2). Interestingly, the aforementioned association was statistically significant when morning cortisol values exceeded normal limits (Figure 3). This finding has not also been presented in the literature, to the best of our knowledge.

Emotional distress before sleep has been linked with increased nightmares and therefore enhanced dream recall [21]. According to our results, increased cortisol levels before sleep may be the cause of this phenomenon. Increased cortisol levels before sleep may offer a favorable substrate for dream formation and eventually better dream recall. However, as presented before, the biological processes affecting dream recall and affect may continue after awakening [9]. Our results come in agreement with the latter suggestion.

Our study failed to show any association between the stage of the menstrual cycle and dream recall/affect. The results of previous studies regarding this subject have been conflicting [22,23]. In a previous study of ours, we showed that, during the luteal phase of the menstrual cycle, women reported pleasant dreams more frequently [19]. However, the sample size of the present study was limited, and thus our results could not be replicated.

Certain limitations of this pilot study must be acknowledged. As mentioned before, the sample size of this study is limited (15 males and 15 females) and the statistical analyses performed may only serve for hypothesis generation. The binary form of the dream recall/affect questionnaire used in our study serves another limitation, as the quality of dreams may be described better using a scale. However, we avoided the use of an elaborate scale due to the pilot nature of our study. Furthermore, possible confounders such as emotional stress were not assessed in this study. Daytime emotions and dreams are bidirectionally associated [24,25]. Nevertheless, subjects with diagnosed mental illness were excluded from our study.

Dream formation, as well as dream recall and affect, are based on complex biological procedures. Our understanding of these phenomena remains obscure. More studies are required to delineate the nature of these processes. The role of hormones such as cortisol in the formation and the ability to recall dreams seems promising, and thus should be further investigated.

## 4. Materials and Methods

For the purposes of this cross-sectional pilot study, 15 male and 15 female volunteers were enrolled. The inclusion criteria included age between 18 and 40 years old and the signed informed consent of the volunteers. More specifically, all volunteers were aged between 25 and 38 years old (mean = 31 ± 4). The age of male participants ranged between 25 and 38 years old (mean = 31 ± 4), while the age of female participants ranged between 25 and 37 years old (mean = 30 ± 4). The exclusion criteria, used during the recruitment of the participants, included the following: (1) history of acute or chronic disease, such as cardiovascular, endocrine, sleep or mental disorder and cancer; (2) use of steroids or antihypertensive medications; (3) pregnancy; (4) irregular menstrual cycle for female participants; (5) shiftwork or night work. All volunteers were instructed to avoid alcohol consumption during sampling days (so alcohol-induced REM stage suppression may be avoided [26]). The study was approved by the Ethics Committee of the Democritus University of Thrace.

Saliva samples were used for the determination of cortisol levels. Male participants were instructed to provide four saliva samples during three consecutive working days. The first sample was obtained between 18:00 and 20:00 on the first day (D1), the second at 10 min after awakening on the second day (D2), the third between 18:00 and 20:00 on D2, and the fourth at 10 min after awakening on the third day (D3). Female subjects followed the aforementioned procedure twice: during the 5th (D1a), 6th (D2a) and 7th (D3a) day, as well as during the 18th (D1b), 19th (D2b) and 20th (D3b) day of the menstrual cycle. Participants were instructed not to eat, drink, smoke or brush their teeth for at least 30 min before sampling. Saliva samples were obtained using Salivettes (Sarstedt^®^, Numbrecht, Germany) and were stored in a refrigerator for approximately 1–2 days until they were transferred to the lab, where they were kept at temperature of approximately −20 °C until analysis. The analysis was performed using electrochemilluminescence (Elecsys Cortisol assay/Cobas, Roche, Basel, Switzerland).

Regarding the assessment of ream recall and affect, each participant was instructed to fill in a questionnaire on D2 and D3, D2a and D3a, as well as D2b and D3b, after awakening. The questionnaire consisted of two binary questions: (1) “Did you have dreams last night?”; (2) “If you did have dreams last night, would you describe them as pleasant or unpleasant?”.

We used the Kolmogorov–Smirnov test to assess whether the distribution of continuous data (age, cortisol levels) is normal. We investigated the possible association of cortisol levels, before and after sleep, with dream recall/affect after awakening, using binary logistic regression analysis. This analysis was performed for each sampling day separately, for each group (males, females on follicular phase, females on luteal phase), as well as for all sampling days collectively, regardless the group of participants. Furthermore, we intended to study whether cortisol levels, exceeding normal limits, after awakening, affect dream recall and content. To do so, we used a cut-off value of 19.1 nmol/L [20], performing the Chi-squared test and Fisher’s exact test, for all the sampling days, regardless of the group of participants. Fisher’s exact test was also used to investigate the possible connection between the phase of menstrual cycle and dream recall/affect, for each day separately (D2a vs. D2b, D3a vs. D3b), as well as regardless of the sampling day (D2a + D3a vs. D2b + D3b). Of the variables/parameters assessed, morning (but not evening) salivary cortisol was normally distributed and also satisfied the linearity predictor/response criterion for implementation of mixed model analysis, with morning salivary cortisol as the dependent variable, gender, age and dream recall or affect as the fixed variables and sampling time as the random effects grouping factor. Evening salivary cortisol, even after logarithmic, square root or inverse (1/x) transformation, was not normally distributed throughout; normality of distribution was noted only in the set of values, after inverse (1/x) transformation, from women in the follicular phase. Bearing this caveat, mixed model analysis, with evening salivary cortisol (after inverse (1/x) transformation) as the dependent variable, gender, age and dream recall or affect as the fixed variables and sampling time as the random effects grouping factor was also implemented. The Statistical Package for Social Science (SPSS Inc, Armonk, NY, USA; version 25.0 for Windows) and JASP v.0.14.1 (JASP Team, University of Amsterdam, Netherlands, 2020) were used for statistical analyses.

## Figures and Tables

**Table 1:** Cortisol levels and dream recall/affect.

Sampling Time	Men		Women (Follicular Phase)		Women (Luteal Phase)	
	Cortisol *: Median (Q1–Q3)	Dream Recall (Positive Affect **)	Cortisol *: Median (Q1–Q3)	Dream Recall (Positive Affect **)	Cortisol *: Median (Q1–Q3)	Dream Recall (Positive Affect **)
**Day 1 Evening**	3.1 (1.5–5.46)	9/15 (8/9)	2.5 (1.9–3.25)	12/15 (8/12)	2.64 (1.5–4.68)	9/15 (3/9)
**Day 2 Morning**	14.88 (11.6–20.45)		13.68 (11.32–23.15)		12.19 (8.46–17.42)	
**Day 2 Evening**	3.03 (1.64–6.06)	7/15 (3/7)	2.89 (1.5–3.71)	11/15 (6/11)	1.98 (1.5–2.89)	10/15 (7/10)
**Day 3 Morning**	15.18 (11.11–19.98)		18.55 (12.82–21.76)		12.63 (9.45–18.5)	

Q1: 1st quartile, Q2: 2nd quartile; * Cortisol levels are measured in nmol/L; ** Represents the fraction of subjects with positive dream recall, who described their dreams as pleasant.

## Data Availability

The data used in this study are available upon request.
